# Analysis of genetic differentiation and genomic variation to reveal potential regions of importance during maize improvement

**DOI:** 10.1186/s12870-015-0646-7

**Published:** 2015-10-24

**Authors:** Xun Wu, Yongxiang Li, Xin Li, Chunhui Li, Yunsu Shi, Yanchun Song, Zuping Zheng, Yu Li, Tianyu Wang

**Affiliations:** Institute of Crop Science, Chinese Academy of Agricultural Science, Beijing, China; Nanchong Academy of Agricultural Sciences, Nanchong, Sichuan China

**Keywords:** Genomic variation, Subpopulation differentiation, *Zea mays* L

## Abstract

**Background:**

Exploring genetic differentiation and genomic variation is important for both the utilization of heterosis and the dissection of the genetic bases of complex traits.

**Methods:**

We integrated 1857 diverse maize accessions from America, Africa, Europe and Asia to investigatetheir genetic differentiation, genomic variation using 43,252 high-quality single-nucleotide polymorphisms(SNPs),combing GWAS and linkage analysis strategy to exploring the function of relevant genetic segments.

**Results:**

We uncovered many more subpopulations that recently or historically formed during the breeding process. These patterns are represented by the following lines: Mo17, GB, E28, Ye8112, HZS, Shen137, PHG39, B73, 207, A634, Oh43, Reid Yellow Dent, and the Tropical/subtropical (TS) germplasm. A total of 85 highly differentiated regions with a D_EST_ of more than 0.2 were identified between the TS and temperate subpopulations. These regions comprised 79 % of the genetic variation, and most were significantly associated with adaptive traits. For example, the region containing the SNP tag PZE.108075114 was highly differentiated, and this region was significantly associated with flowering time (FT)-related traits, as supported by a genome-wide association study (GWAS) within the interval of FT-related quantitative trait loci (QTL). This region was also closely linked to *zcn8* and *vgt1*, which were shown to be involved in maize adaptation. Most importantly, 197 highly differentiated regions between different subpopulation pairs were located within an FT- or plant architecture-related QTL.

**Conclusions:**

Here we reported that 700–1000 SNPs were necessary needed to robustly estimate the genetic differentiation of a naturally diverse panel. In addition, 13 subpopulations were observed in maize germplasm, 85 genetic regions with higher differentiation between TS and temperate maize germplasm, 197 highly differentiated regions between different subpopulation pairs, which contained some FT- related QTNs/QTLs/genes supported by GWAS and linkage analysis, and these regions were expected to play important roles in maize adaptation.

**Electronic supplementary material:**

The online version of this article (doi:10.1186/s12870-015-0646-7) contains supplementary material, which is available to authorized users.

## Background

Maize (*Zea mays* L.) is widely planted throughout the world, including in more than 70 countries across six continents [1]. Maize originated in south-central Mexico [2] and spread throughout the Americas for thousands of years before it was introduced to Europe, Africa, and Asia after Columbus discovered the New World [3]. During this spread, maize continually improved via natural and artificial selection in order to adapt to different environments [4]; a number of landraces and inbreds were developed [5], and many hybrids with high yields have been released to satisfy the increasing need of humans [6].

In the past several decades, maize’s diffusion [3], improvement [7–11], pedigrees [12, 13], and genetic basis for phenotypic variations [14–16] have been well documented, providing scientific proof for the genetic contributions to historical yield increases and the formation of heterotic groups. For instance, American maize germplasms were divided into three main heterotic groups: Iowa Stiff Stalk Synthetic (SS), Non-Stiff Stalk (NSS), and Iodent (IDT) [10]. African maize germplasms were divided into three clusters: Meso-American landraces, Coastal Brazilian landraces, and Tropical varieties [17]. Chinese maize germplasms are divided into five subpopulations: including Ludahonggu, TSPT, P group, Reid and Lancaster [18–20]. The aforementioned studies provided useful information for both heterosis utilization and the dissection of the genetic basis for complex traits, but the accessions used in previous studies were obtained from a single geographical origin and have relied on the smallest number of markers, which limits our understanding of genetic differentiation. The development of high-throughput genotyping strategies has facilitated the study of historical genetic changes in maize [21–23]. Recently, another large natural panel of 2,815 maize accessions was investigated using the genotyping by sequencing (GBS) method [12], and this study provided abundant information about pair relationships of accessions and identified many new genetic loci associated with flowering time (FT)-related traits. Five subpopulations were observed in this paper; the distance between SS and NSS subpopulations was small, which indicated a slight bias when comparing with previous studies and the knowledge of maize pedigrees based on breeding practice [10, 22, 24–26].

In addition, many studies of genomic variation reported using G_ST_ and its relatives (D_EST_, F_ST_) [27]. Haag et al. [28] demonstrated that D_EST_ constituted an alternative measure of genetic differentiation between populations. However, the traditional F_ST_ value has been widely used to estimate plant genetic differentiation. A higher F_ST_ of selected features was observed between subpopulations using 284 maize inbreds from Minnesota [22], and this value was larger than that between temperate maize germplasms [9]. Romay et al. [12] showed that most of germplasms from classic breeding programs of the Corn Belt were closely related, with an average pair-wise F_ST_ of 0.04, which was larger than the 0.027 value reported between tropical and temperate lines [29] and the 0.02 value reported between landraces and improved lines. Nevertheless, this value did not exceed the 0.11 value reported between teosinte and landraces [30] However, most studies have previously only reported the differentiation phenomenon and extent of genetic variation between subpopulations. The potential genomic regions of importance that are highly differentiated and associated with putative function are poorly understood, especially for maize.

In this paper, we integrate maize germplasms from America, Africa, Europe and Asia, including 1857 accessions from more than sixteen countries worldwide, and present an in-depth analysis of genetic differentiation and genomic variation using a dataset of 43,252 single-nucleotide polymorphisms (SNPs). We dissected the genome-wide variation patterns of selection fixation, uncovered the subdivision of population structure, identified highly differentiated genomic regions between subpopulations, combined genome-wide association studies (GWAS) of FT-related traits, and compared the results with public data on the quantitative trait loci (QTL) and bioinformatics analysis to identify adaptive genomic regions and relevant candidate genes that may have been important during maize development and the formation of modern heterotic groups.

## Results

### Ascertainment bias

The average correlation coefficients of the first five principal components (PCs) between one given subset and the entire set with all markers are shown in Additional file 1: Figure S1. The correlation coefficients between the subset and the entire set sharply increased from 0.65 for a marker number of 500 to 0.97 for a marker number of 700. A second sharp increase emerged when the marker number increased from 800 to 1000, with a corresponding increase in the correlation coefficient from 0.97 to 0.99. Furthermore, the correlation coefficient did not significantly change when the marker number increased from 2000 to 43,252. The results indicated that 1,000 SNPs might be sufficient for population structure analyses.

### Model-based population structure

**Fig. 1:**
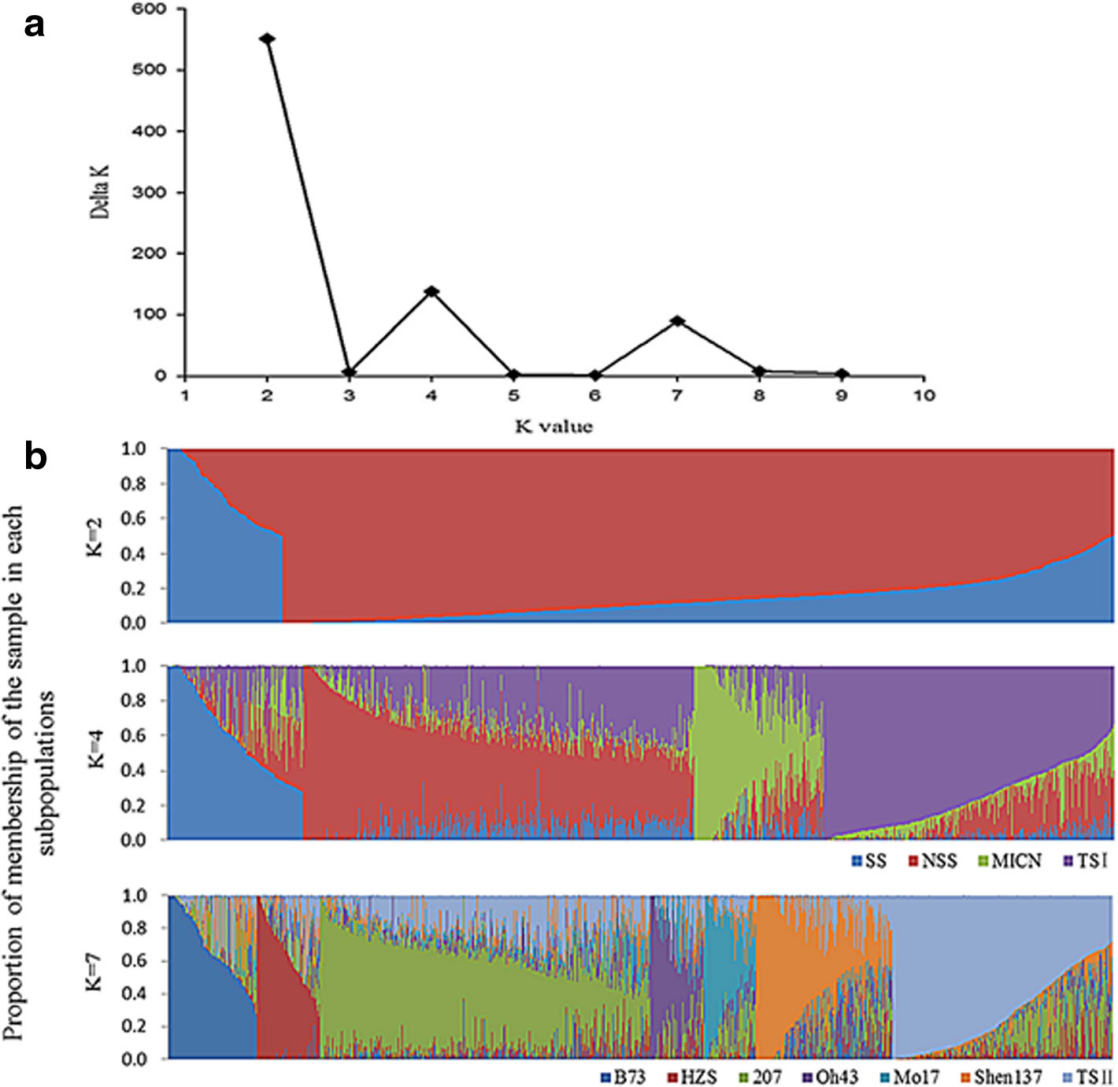
Model-based subdivision of population structure. ‘**a**’ presents the estimation of the Ln (probability of data). Delta K was calculated from K = 2 to K = 9. ‘**b**’ presents the population structure of the 1,857 maize accessions deduced by membership coefficients (Q values). Each horizontal bar presents one accession, which is consisted of K colored segments. ‘SS’ is the abbreviation of Stiff Stalk Synthetic group, “MICN” Modified Introduction of China, ‘TS’ Tropical/Subtropical group, and NSS Non-Stiff Stalk

The subpopulations of 1857 accessions based on the admixture model-based algorithm were analyzed in depth using the even distribution of 5000 SNPs. The results are depicted in Fig. 1. The delta K (∆K) peak was maximized when k = 2 (Fig. 1a), indicating that the accessions could be categorized into two groups: tropical/subtropical (TS) germplasm and temperate germplasm (Fig. 1b k = 2). A second peak of ∆K emerged at k = 4 (Fig. 1a), indicating that this panel could be further divided into four subgroups: SS, NSS, Modified Introduction in China (MICN), and TS I (Fig. 1b k = 4). Notably, MICN formed during the long history of maize breeding in China because Chinese maize breeders have developed a number of inbred lines derived from Chinese landraces and U.S. hybrids. These varieties significantly differ from U.S. inbreds [19]. A third peak of ∆K was observed at k = 7 (Fig. 1a), indicating that this panel could be comprehensively categorized into seven subpopulations, each including one of the following representative lines: B73, Huangzaosi (HZS), 207, Oh43, Mo17, Shen137, and some from TS regions (Fig. 1b, k = 7). Detailed information for each accession is listed in Additional file 2: Table S1.

### Clustering analysis

**Fig. 2:**
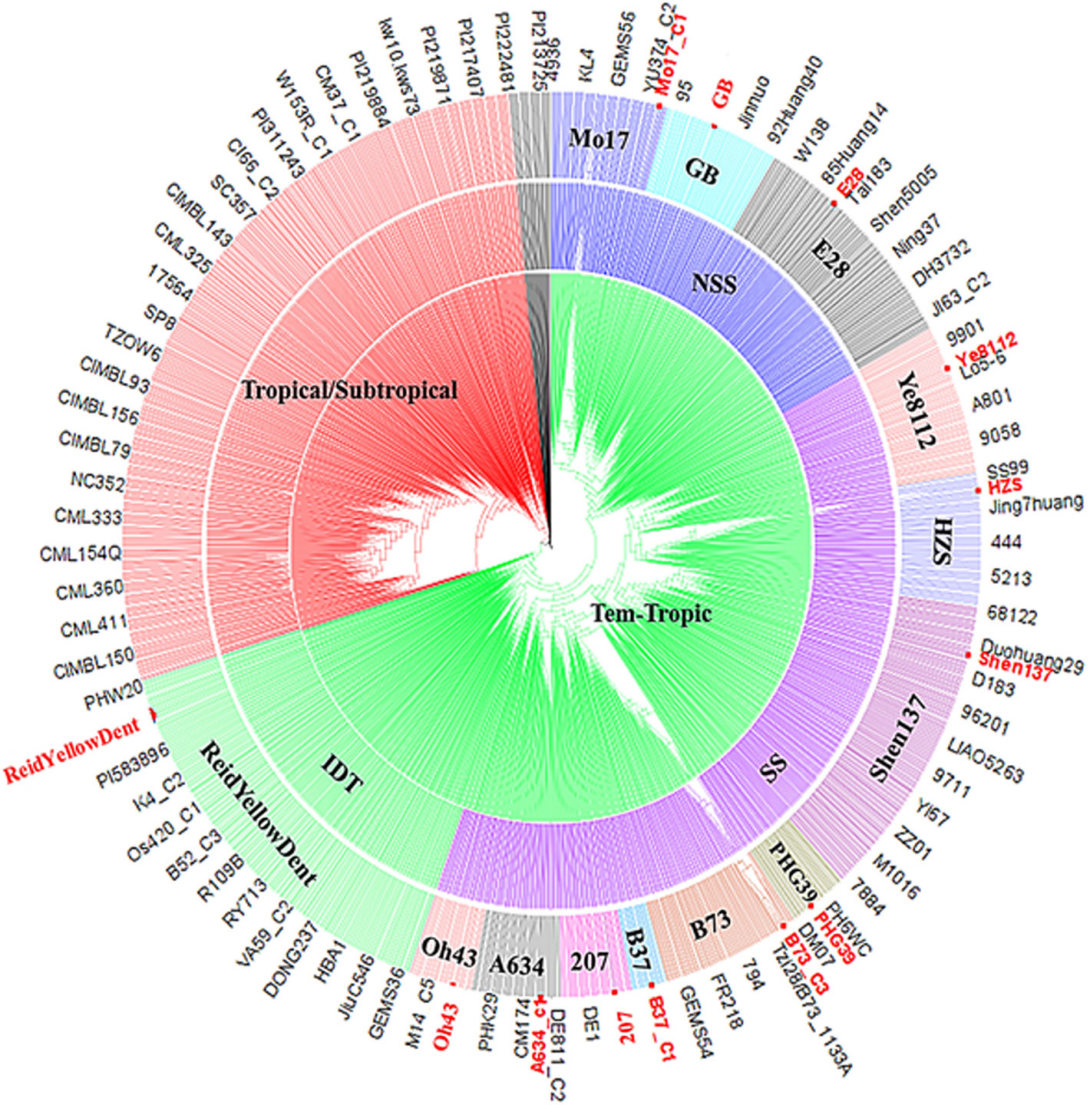
Neighbor-joining trees of the 1,857 maize accessions. Mo17 is a representative line of Non-Stiff Stalk (NSS). GB is a representative line derived Chinese landrace. E28 is a representative line of the Ludahonggu group. Ye8112 a representative line of the Modified Reid group. ‘HZS’ is an abbreviation of Huangzaosi, which is a representative line of the Tangsipingtou group (TSPT). Shen137 is a representative line of the PA group. PHG39 is a parent derived from Argentine Maize Amargo background. B73 is a representative line of Stiff Stalk Synthetic (SS). B37, 207, A634, Oh43, and Reid Yellow Dent are the representative lines of different subpopulations, respectively

A neighbor-joining tree was constructed based on the modified Euclidean distance and is shown in Fig. 2. The 1857 accessions were clustered into two major groups according to their origins: the TS and Tem-tropic subpopulation. The TS subpopulation contained 525 accessions, including 195 accessions from Mexico, 187 from the U.S., 77 from China, 17 from Sudan, 10 from Thailand, 9 from Canada, 9 from Tanzania, 6 from Nigeria, 3 from Somalia, 3 from Benin, 3 from Zambia, 3 from Chad, 2 from Spain, 1 from Ghana, 1 from Germany, 1 from Yugoslavia, and 1 from Egypt (Additional file 2: Table S1). The Tem-tropic subpopulation contained 1,332 accessions, which could be further clustered into four subpopulations, SS, NSS, Iodent (IDT) and TS, according to their origins and pedigrees. A further analysis showed that the accessions from these four subpopulations could be clustered into 13 subgroups, with the following representative lines: Reid Yellow Dent, Oh43, A634, 207, B37, B73, PHG39, Shen137, Huangzaosi (HZS), Ye8112, E28, GB and Mo17 (Fig. 2).

### Principal component analysis (PCA)

**Fig. 3:**
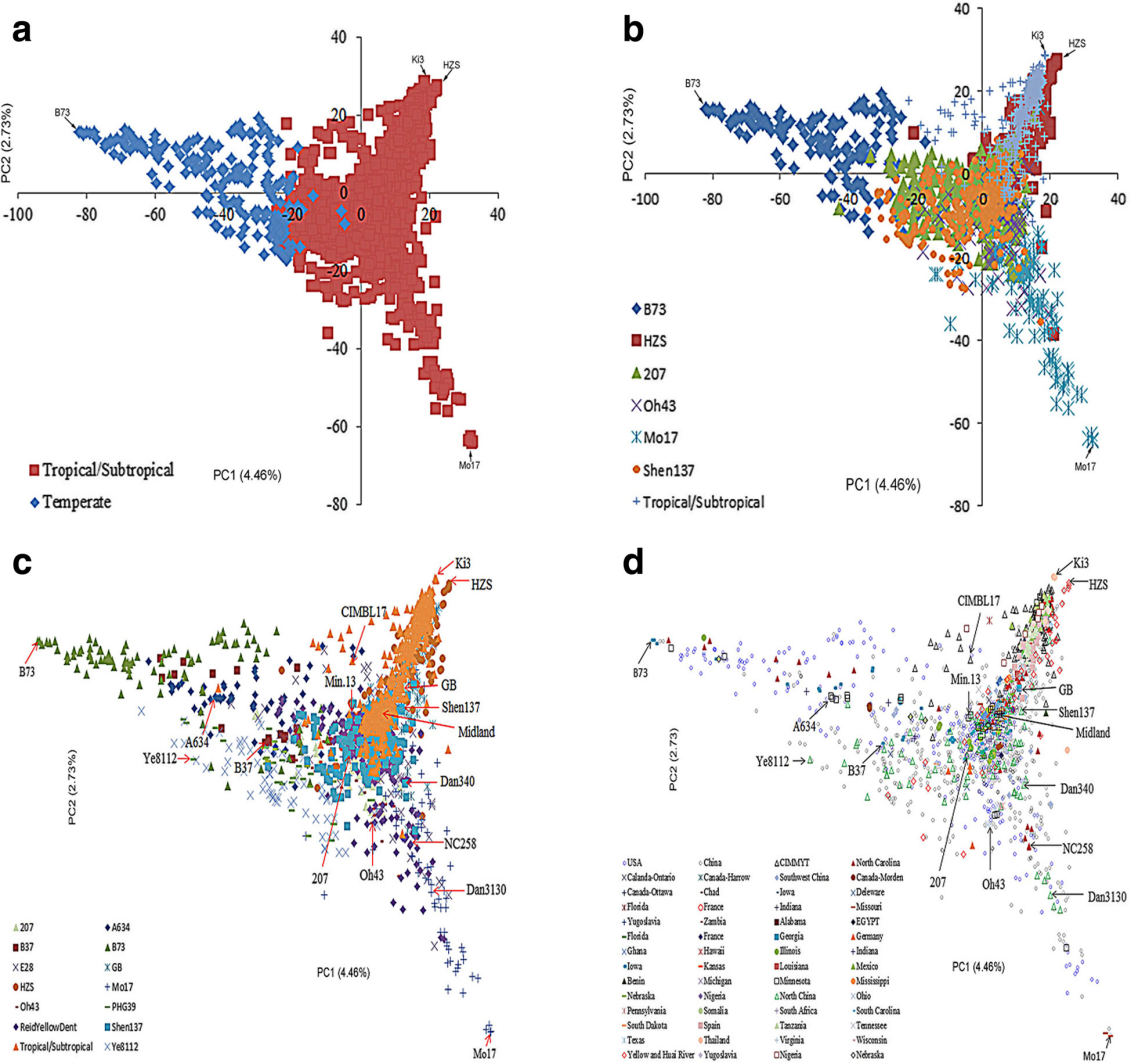
Results of principal components (PCs). Plots ‘**a**’ and ‘**b**’ show the comparison between the model-based population structure and the PC analysis results. Plot ‘**c**’ shows the comparison between the PC analysis results and the N-J tree constructed based on modified Euclidean distance. Plot ‘**d**’ shows the comparison between the original information and the PC analysis results

The PCA results showed comprehensive patterns of subpopulation and a good agreement with both model-based population structure and clustering analyses (Fig. 3). The entire panel of 1857 accessions exhibited moderate differentiation and some overlap between the temperate and TS germplasm; representative lines from the TS and temperate region significantly differed, e.g., B73 from the temperate and Ki3 from the TS region of Thailand, but the accessions from the adjacent regions did not markedly differ. Which may be resulted by the lager introgression existing between temperate and tropical/subtropical accessions and lower power of PCA in population structure analysis by using only two PCs. The accessions from the temperate subpopulation were further categorized into the B73 subpopulation according to the results of model-based structure analysis (Fig. 3b) or the Ye8112, B37 and A634 subpopulations based on the results of modified Euclidean distance (Fig. 3c). Based on the pedigrees, most lines were from the U.S. and China (Fig. 3d and Additional file 2: Table S1). In addition, the TS population was further divided into the HZS, 207, Oh43, Mo17 and Shen137 subpopulations based on the model-based population structure, which corresponded to HZS, GB, Shen137, Mo17, and Reid Yellow Dent based on a clustering analysis (Fig. 3c). These subpopulations contained inbred lines of a TS lineage in their pedigrees or lines from CIMMYT, Mexico and other tropical regions (Fig. 3a and d). Moreover, many accessions were categorized into new groups, such as the PHG39, 207, A634, Oh43, B37 and E28 subpopulations; most accessions in these groups originated from regions between temperate and TS zones (Fig. 3) due to the introgression of TS genotypes into regions of temperate germplasms.

### Summary statistics of genetic diversity

**Fig. 4:**
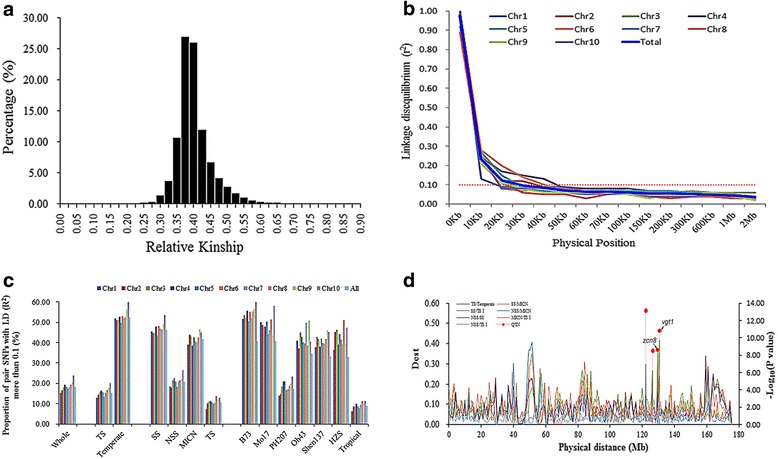
Summary statistics of genetic variation existing in the whole set of accessions. ‘**a**’ is a picture of pair-wise kinship of the 1857 accessions. ‘**b**’ displays the decay level of linkage disequilibrium (LD) on different chromosomes and across the whole genome. ‘**c**’ shows the comparison of LD level between different subpopulations. ‘**d**’ pictures the genomic differentiation on Chromosome 8

The accessions of the entire panel of 1857 accessions were moderately similar, with more than 96.22 % of the pair-kinship coefficients varying from 0.30 to 0.53 (Fig. 4a). The average linkage disequilibrium (LD) distance was 30 kilo-bases (kb), varying from 20 to 50 kb, with an r^2^ exceeding 0.1 (Fig. 4b). Combining the results of both the model-based population structure and genomic variation analyses indicated pronounced patterns of genetic variation among different subpopulations. These patterns were fixed by artificial or natural selection and resulted in the division of subpopulations during breeding. The TS subpopulation was more genetically diverse than the temperate subpopulation, with gene diversities (GDs) of 0.364 and 0.284, respectively, and polymorphism information contents (PICs) of 0.281 and 0.231, respectively (Table 1). Similar trends were validated with a smaller proportion of SNPs in LD for TS when comparing with a larger proportion of SNPs in LD for the temperate subpopulation (Fig. 4c).

**Table 1:** Summary statistics of genetic diversity

Index	Total	K = 2		K = 4				K = 7						
		TS	Temperate	SS	NSS	MICN	TS I	B73	Mo17	PH207	Oh43	Shen137	HZS	TS II
Gene Diversity	0.365	0.364	0.284	0.301	0.361	0.306	0.348	0.268	0.299	0.360	0.294	0.311	0.272	0.345
PIC	0.291	0.291	0.231	0.244	0.289	0.247	0.279	0.219	0.242	0.287	0.239	0.250	0.223	0.277
Heterozygosity	0.046	0.048	0.025	0.027	0.058	0.047	0.037	0.023	0.033	0.065	0.028	0.049	0.034	0.033

Note: K is the number of subpopulations. ‘TS’ is an abbreviation of Tropical/Subtropical subpopulation. ‘SS’ is an abbreviation of Stiff Stalk Synthetic subpopulation. ‘NSS’ is an abbreviation of Non-Stiff Stalk. ‘MICN’ is an abbreviation of Modified Introduction of China

### Genomic differentiation between subpopulations

**Fig. 5:**
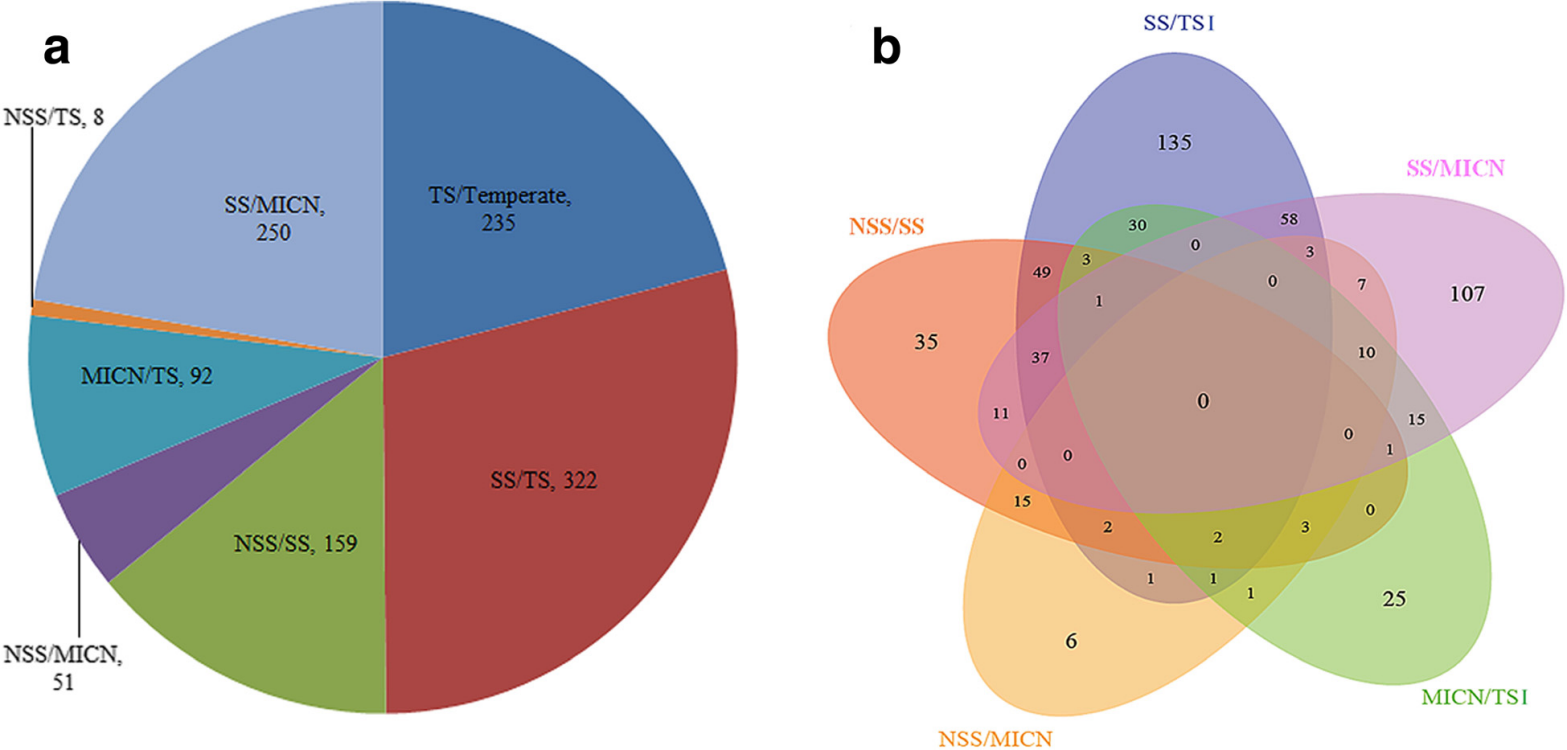
Counts of genetic regions with high differentiation. ‘**a**’ shows the counts of genomic regions for each subpopulation pair. ‘**b**’ shows the comparison of genomic regions with high differentiation among different subpopulation pairs

The proportion of genetic variance due to subpopulations (D_EST_) was measured to interpret the genomic variation between subpopulations (Table 2, Fig. 4(d), Fig. 5 and Additional file 1: Figure S2). The D_EST_ indicated different patterns of genomic differentiation between the subpopulations, ranging from 0 to 0.39 between TS and Temperate (average 0.08), from 0 to 0.45 between TS I and SS (average 0.09), from 0 to 0.45 between SS and NSS (average 0.07), from 0 to 0.41 between NSS and MICN (average 0.05), from 0 to 0.38 between MICN and TS I (average 0.06), from 0 to 0.30 between NSS and TS I (average 0.03), and from 0 to 0.57 between SS and MICN (average 0.08). The SS and TS I varieties were more differentiated, with 332 genomic regions having a large D_EST_ (twice the average level) (Fig. 5a). Furthermore, 250 genomic regions were highly differentiated between SS and MICN, 235 were highly differentiated between TS and Temperate, 92 were highly differentiated between MICN and TS I, 51 were highly differentiated between NSS and MICN, and 8 were highly differentiated between NSS and TS I, with a D_EST_ of more than twice the average level. Most importantly, 85 highly differentiated regions with a D_EST_ exceeding 0.2 were identified between the TS and the temperate subpopulations. Of these 85 regions, 68 were located within the interval of plant architecture or FT-related QTL, and two regions were closely linked to *vgt1* and *zcn8* (Additional file 2: Table S2 and S3). Furthermore, a number of special genomic regions were also found to be highly differentiated. In particular, subpopulation pairs and common regions were identified among different population pairs (Fig. 5b). In total, 303 genomic regions with a high D_EST_ of more than 0.2 were detected, and these regions were located within 197 FT- or plant architecture-related QTL. For example, the region containing the tag SNP PZE.108075114 differed more between the TS and temperate subpopulations and was associated with a D_EST_ of 0.32; this region was located within an FT-related QTL cluster and contained the flanking markers PHTi060 and bnlg1599 (Additional file 2: Table S3).

**Table 2:** Variation of D_EST_ between subpopulations

K = 2	Temperate	TS					
Temperate	0.000	0.170					
TS		0.000					
K = 4	SS	NSS	MICN	TS I			
SS	0.000	0.164	0.146	0.172			
NSS		0.000	0.134	0.053			
MICN			0.000	0.121			
TS				0.000			
K = 7	207	B73	HZS	Mo17	Oh43	Shen137	TS II
207	0.000	0.251	0.283	0.260	0.242	0.113	0.059
B73		0.000	0.295	0.309	0.246	0.202	0.247
HZS			0.000	0.242	0.194	0.199	0.208
Mo17				0.000	0.180	0.182	0.226
Oh43					0.000	0.156	0.194
Shen137						0.000	0.110
TS II							0.000

### Genome-wide study of FT-related traits

**Fig. 6:**
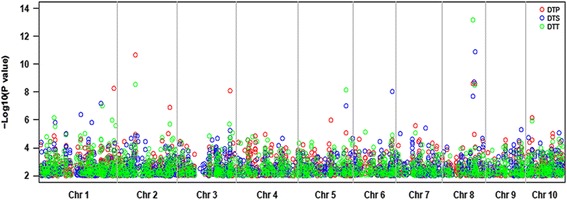
Manhattan plot of GWAS results for flower time related traits. Red cycle refers to days to pollen-shedding (DTP), blue cycle shows days to silking (DTS), and green cycle shows days to tasseling (DTT). Red line shows the cutoff value of 5.94 (defined as: −log_10_ (0.05/43,252))

The phenotypes of FT-related traits were significantly positively correlated between the environments (Additional file 1: Figure S3). Thus, the BLUPs for each accession across the three environments were calculated, and the phenotype-genotype associations were analyzed. To validate the putatively adaptive function of highly differentiated target regions, we used the FT-related traits DTT, DTS, and DTP to perform a GWAS with 43,252 SNPs as a case study. The results indicated that some highly differentiated genomic regions were associated with FT-related traits. For example, the SNP of PZE-108070380 was significantly associated with DTT (*P* = 7.05 × 10^−14^), DTP (*P* = 2.57 × 10^−9^) and DTS (*P* = 2.12 × 10^−8^) (Fig. 6). This SNP was located within the *zcn8* gene, which is involved in maize migration from tropical to temperate regions [31]. The SNP PZE-108076585 was significantly associated with DTS (*P* = 1.43 × 10^−11^) (Fig. 6). This SNP was located within the *vgt1* gene, which is involved in maize adaptation [32]. Furthermore, twelve other SNPs were also strongly associated with FT-related traits (Fig. 6), and the regions surrounding these SNPs were more differentiated than the rest of the genome (Fig. 4d, Additional file 1: Figure S2, Additional file 2: Table S2).

## Discussion

### Moderate SNPs are reliable in interpreting population structure division

Previous reports compared the effect of different marker systems and concluded that the subdivision of populations depended on the marker size and population [18, 33–35]. For instance, when 884 SNPs were used in one association panel of 154 inbred lines, more than 26.4 % of lines were allocated to the mixed group. This rate was higher than the 20.6 % rate identified by using 84 simple sequence repeat (SSR) markers [35]. Comparing the effect between 847 SNPs and 89 SSRs in one panel of 254 inbred lines yielded similar results [36], they proposed that many more SNPs would be required to study population structure. Here, we compared the average correlation coefficients of division for subpopulations between one given subset with different marker sizes and the entire set with all markers; we used SNPs varying from 500 to 43,252 in a panel of identical samples. The results showed that 700 SNPs are sufficient to reliably divide subpopulations in this panel, with an average correlation coefficient of the first five PCs of 0.97 between the subsets and the entire set of SNPs. The average correlation coefficient could be increased to 0.99 by increasing the number of SNPs to 1000 (Additional file 1: Figure S1). Yu et al. [37] reported moderate genetic diversity with a PIC of 0.24 for a sample size of 274. We herein report a similar PIC of 0.29 for a sample size of 1,857. Yu et al. [37] demonstrated that more than 1000 SNPs are necessary needed to robustly estimate the genetic differentiation of a naturally diverse panel, and this requirement exceeded the 700–1000 SNPs found to be necessary herein. Thus, a larger sample size is expected to significantly improve the detection power of subdivisions in the populations. These results were consistent with those reported by Morin et al. [33], who compared the subpopulation differentiation for sample sizes ranging from 10 to 100. The results reported herein suggested that a moderate SNP marker number (700–1000) is sufficient to divide population structures in this panel.

### Comprehensive patterns of population structure in maize inbreds worldwide

The analysis of population structure is an important step in dissecting the genetic basis of complex traits via association analyses [38]. Such an analysis can result in false positive errors [34]. In the last several decades, a number of studies have evaluated the population structure of specific germplasms using limited sample sizes and sources. These studies independently provided specific information about the subpopulation differentiation of approximately 600 Minnesota maize germplasms [22], 172 Dent germplasms from Hohenheim [39], 400 maize lines from North America [23], 367 elite lines from China [19] and 527 lines representing TS and temperate backgrounds [40]. Here, we integrated maize germplasms from America, Africa, Europe and Asia, including 1857 accessions from more than 16 countries worldwide, to investigate subpopulation differentiation. The outputs of STRUCTURE V2.3.3 identified seven subpopulations: including B73, HZS, 207, Oh43, Mo17, Shen137, and TS II (Fig. 1). These results provided much more information about maize subpopulation differentiation than previous studies. In fact, the B73 (SS), Mo17 (NSS), Oh43, and 207 (IDT) subpopulations were identified using SSR markers and an Illumina MaizeSNP50 Beadchip [22]. HZS (TSPT), Shen137 (PA derived from Pioneer hybrid 78599), and TS I subpopulations were also identified in previous reports [18, 19, 41]. In addition, the findings this study was also consistent with known pedigrees. For example, LH61 shared 87.5 % of its nuclear genetic material with Mo17 [42] and clustered into the Mo17 subpopulation with an ancestry membership of 0.91 (Additional file 2: Table S1). These results were consistent with those reported by Lorenz et al. [42]. Furthermore, the clustering analysis identified many more clusters, including Mo17, GB, E28, Ye8112, HZS, Shen137, PHG39, B73, B37, 207, A634, Oh43, and Reid Yellow Dent (Fig. 2). The identification of these clusters indicated that our clustering analysis increased the resolution of the categorization of accessions into subpopulations compared with the model-based method, which commonly identifies six subpopulations, Mo17, B73, HZS, Oh43, 207, and Shen137. For instance, PB80 and A632 shared 75 % and 93.75 % of the nuclear genetic material of B73 and B14, respectively [42], these two lines clustered into the same subpopulation as B73 and B14, respectively. This clustering was consistent with a report by Lorenz et al. [42]. Most importantly, the clustering analysis in this study identified new subpopulations that are represented by the following lines: GB, E28, Ye8112, PHG39, B37, A634, and Reid Yellow Dent. These lines correspond to the following heterotic groups: Chinese Landrace (GB) [19], Ludahonggu (E28) [41], PB (Ye8112, B37) [19] derived from modern U.S. hybrids, Commercial hybrid-derived lines (PHG39, A634) [10], and U.S. landrace (Reid Yellow Dent) [10], respectively. Of these groups, Chinese Landrace is mainly distributed in the northeast and southwest of China, and this variety originated from the North-American Mid-West and Mexican highlands, respectively [3]. These landraces yielded new subpopulations and have been widely used in maize-breeding programs [19]. For example, E28 is a representative line derived from crossing the landrace Ludahonggu with modified introduction lines according its pedigree [19]. Ye8112 was selected from the hybrid “8112”, which originates in the U.S. [41]. Some of the lines were derived from this line, such as Ye478 and 488, which were clustered in the heterotic group of PB [19, 41]. A634 was derived from the MN13 lineage [22], is highly utilized in U.S. hybrid maize breeding. This line constituted 4.2, 7.8, and 3.0 % of the total U.S. seed requirement in 1970, 1975, and 1979, respectively, and lots of lines were derived from A634 [13]. B37 is an important public line that was widely used to develop Pioneer hybrids during the 1980s [6]. The selection of a second cycle line from Pioneer hybrids resulted in new lines, which formed a subpopulation represented by B37. PHG39 is a representative inbred Maize Amargo germplasm line from which many protected corn lines have been developed. Furthermore, several important first cycle recombinant lines derived from PHG39 have been considered for commercial maize breeding [10]. These results provide maize breeders with more definitive information to effectively use historical genetic resources while maintaining the heterotic patterns necessary for hybrid breeding.

### Genomic differentiation and putative functions

Genomic differentiation between subpopulations is a fundamental challenge in population genetics. Maize originated in tropical central-Mexico and rapidly spread to colder, temperate regions worldwide [32]. This diffusion caused maize to adapt to local environments by developing traits that allowed it to thrive in these environments, i.e., changes in FT and plant architecture. These changes allowed maize to reach maturity within different growing seasons. Some studies have documented the pair-wise F_ST_ between subpopulations while considering genomic differentiation [9, 43, 44]. Schaefer and Bernardo [22] reported an average pair-wise F_ST_ of 0.165 for one diverse panel of 284 maize inbreds; this value ranged from 0.054 between the A321 and Oh43 subpopulations to 0.325 between the Mo17 and B73 subpopulations. Romay et al. [12] found that most germplasms from classic breeding programs of the Corn Belt were closely related, with an average pair-wise F_ST_ of 0.04. However, the differentiation regions and putative function remain poorly understood. Moreover, the D_EST_ was also demonstrated as a measure genomic differentiation. This parameter relies on the genotypic rather than allelic number and is corrected for heterozygosity [27]; values close to zero indicate little differentiation, and values close to unity indicate nearly complete differentiation. Therefore, the D_EST_ was used in the present study to evaluate the genomic variation between the subpopulations, and the results of this analysis revealed strong differentiation among the subpopulations. This differentiation was attributed to the continuous fixation of target genomic regions within subpopulations and strong isolation between subpopulations during maize breeding practices. The pair-wise D_EST_ between the TS and the temperate subpopulations was 0.17 (Table 2), and 235 highly differentiated genomic regions were identified (Fig. 5). Most adaptive traits were selected and fixed during maize’s long evolution and adaptation from tropical to temperate climates [31]. This fixation caused the high genomic differentiation between TS and temperate germplasms (Table 1, Figs. 2 and 3). Interestingly, 85 strongly differentiated genomic regions with a D_EST_ exceeding 0.2 were identified between the TS and the temperate subpopulations. A genetic analysis showed that these 85 regions comprise 79 % of the genetic variation of this panel (Additional file 1: Figure S4). Of these regions, 15 were significantly associated with FT-related traits based on GWAS (Fig. 4d and Additional file 1: Figure S2). In addition, two significant QTNs were closely linked to *zcn8* and *vgt1* (Fig. 4d), which are involved in maize migration and adaptation from tropical to temperate climates [31]. Beyond that, 66 highly differentiated regions were located within the interval of plant architecture or FT-related QTL (Additional file 2: Table S3). In addition, 159 highly differentiated genomic regions were also identified between SS and NSS subpopulations, with a D_EST_ exceeding 0.16 (Fig. 5). Furthermore, 15 regions located within FT- or plant architecture-related QTL were also identified (Additional file 2: Table S3). This finding was consistent with the marked distance between SS and NSS (Figs. 1, 2 and 3). SS and NSS are two major heterotic groups used in U.S. breeding programs that are represented by the lines B73 and Mo17, respectively. Previous studies also reported a significant distance between these two groups [23]. Furthermore, other highly differentiated genomic regions between specific subpopulation pairs were also identified, and these regions were located within a number of QTLs associated with FT- or plant architecture-related traits mapped using different bi-parental populations (Additional file 2: Table S3). In total, 303 genomic regions with a high D_EST_ of more than 0.2 were detected, and these regions were located within 197 FT- or plant architecture-related QTLs. For example, the region containing the tag SNP PZE.108075114 was more differentiated between TS and the Temperate subpopulations (D_EST_ = 0.32), and this region was located within one FT-related QTL cluster that contained the flanking markers PHTi060 and bnlg1599. These results indicate genomic regions of interest for the formation of given subpopulations and provide new insight into the dissection of the genetic basis of complex traits.

## Conclusions

Here we reported that 700–1000 SNPs were necessary needed to robustly estimate the genetic differentiation of a naturally diverse panel. In addition, 13 subpopulations were identified based on genotyping and pedigree information. On this base, 85 genetic regions with higher differentiation between TS and temperate maize germplasm, and 197 highly differentiated regions between different subpopulation pairs were identified, which contained some FT- related QTNs/QTLs/genes supported by GWAS and linkage analysis, and some known genes of *vgt1* and *zcn8* associated with maize adaptation from tropical to temperate belts, were also included in these regions. Therefore, we concluded that these differential regions were expected to play important roles in maize adaptation. These results would provide abundant information on the differentiation of subpopulations and new insight to help dissect the genetic basis of complex traits.

## Methods

### Plant materials

The present study involved an integrated diverse natural panel of 1857 accessions collected from around the world, including 400 accessions from the U.S. Department of Agriculture (USDA)’s National Plant Germplasm System [23], 280 from the North Central Regional Plant Introduction Station of the USA [45], 368 from CIMMYT [21], 48 from Africa [17], and 890 from the institute of crop sciences of the Chinese academy of agricultural sciences (ICS/CAAS). Chinese germplasm contained two sets of inbred lines: one from a previously established core [46], of 242 diverse inbred lines historically used in Chinese maize breeding and another of recently collected lines from research institutions or companies. This latter category included 648 elite inbred lines that are primarily used in current maize breeding [19]. Detailed information is listed in Additional file 2: Table S1.

### Phenotypic evaluation

The FT-related traits of 1176 out of 1857 accessions were evaluated in three environments, including Beijing in 2014 (spring-sowing), Xinxiang in Henan Province in 2014 (summer-sowing), and Gongzhuling in Jilin Province in 2014 (spring-sowing). At each location, accessions were planted based on a randomized experimental design. Plants (15 plants/row) were sown in single rows that were 4 m long and separated by a distance of 0.6 m. The plant density was 52,400 plants per hectare, and experiments were conducted in duplicate. FT-related traits included days to tasselling (DTT), days to silking (DTS), and days to pollen-shedding and were recorded when 50 % of plants exhibited the corresponding traits. An ANOVA was performed using the PROC GLM model. A Pearson correlation analysis of FT-related traits across different environments was calculated using the PROC CORR model. The best linear unbiased predictor (BLUP) calculation was implemented using a PROC MIXED model, with genotype, location, genotype by location, and replications as random effects [47]. All above analyses were completed using the SAS software (Release 9.3; SAS Institute, Cary, NC).

### Genotyping datasets

The 523 newly collected inbred lines were genotyped using a MaizeSNP50 BeadChip and 56,110 SNPs (http://support.illumina.com/array/array_kits/maizesnp50_dna_analysis_kit.html). When maize seedlings were one month old, the leaves of five plants were sampled in bulk to extract genomic DNA according to the modified CTAB procedure [48]. The quality of the DNA was assessed and the DNA was genotyped at the Beijing Compass Biotechnology Company according to the Infinium® HD assay ultra-protocol guide. In addition, the SNP genotyping datasets of the other accessions were extracted from public datasets, including those of 400 accessions submitted by van Heerwaarden et al. [23], 48 African accessions submitted by Westengen et al. [17], 368 CIMMYT accessions submitted by Li et al. [21], 280 accessions submitted by Flint-Garcia et al. [45], and 367 elite lines submitted by Wu et al. [19]. Finally, all genotypes from different panels were integrated according to the identical physical position and markers names. Alleles forms were transformed based on the pair wise base complementary. Then 43,252 SNPs were successfully obtained for the 1,857 accessions according to the following SNP screening criteria: (1) the minor allele frequency (MAF) exceeded 0.05, (2) the missing rate is less than 0.2, and (3) the position of the marker is unambiguous on a physical map.

### Ascertainment bias of SNPs and PCA

To evaluate the ascertainment bias of SNPs for evaluating the subdivision of population structure, different sample sets of SNPs were sampled across 43,252 SNPs, with window size varying from 50 kb to 0.2 Mb, wherein 500, 700, 800, 1000, 2000, 5000, 10,000 and 15,000 SNPs with highly genetically diverse, low missing rates, and evenly distributed across the genome were selected to do population structure analysis The subdivision of population structure for this panel was deduced with a PCA according to the method described by Patterson et al. [49] using the TASSEL software 5.0 [50]. The correlation PCs was analyzed using the SAS software (Release 9.3; SAS Institute, Cary, NC). Additionally, the average correlation coefficient of the first five PCs was used to deduce the bias extent of one given subset based on the subdivision of population structure.

### Model-based population structure analysis

According the comparison of population subdividing based on different sample sets of SNP markers. A total of highly genetically diverse 5000 SNPs with low missing rates and evenly distributed across the genome were selected to estimate the population structure of the 1857 accessions using a model-based approach [41] in STRUCTURE V2.3.3 [34]. The K value (the number of subpopulations) ranged from 1 to 10, and five runs were completed for each K; the burn-in period was 5000, and 5000 replications were completed. The adhoc statistic delta K (∆K) was used to determine the optimum number of subpopulations [51]. The outputs of STRUCTURE were integrated using CLUMPP software [52]. Subpopulation assignments were based on maximum membership probabilities for each accession [22].

### Neighbor-joining tree construction

To obtain an in-depth picture of the genetic relationships between the 1857 accessions with contrasting origins, the genetic distance between accessions was calculated using 43,252 SNPs based on the modified Euclidean distance [53], which was defined as follows: D = 1 - identity by state (IBS) similarity, and the IBS was the probability that alleles derived at random from two individuals at identical loci are the same. For any two accessions, the probability of IBS was averaged over all non-missing loci. A cladogram was then constructed using the distance matrix described above based on the neighbor-joining (NJ) algorithm [54]. Clusters were identified from the resultant phylogenetic tree. All of the above calculations were conducted using the TASSEL software 5.0 [50].

### Basic genetic statistics

Pair relatedness among the 1,857 accessions was estimated by definition as follows: F_ij_ = (Q_ij_ – Q_m_/(1 – Q_m_), where Q_ij_ is the probability of IBS for random loci from i and j, and Q_m_ is the average probability of IBS for loci from random individuals. Pair-kinship coefficients of 0, 0 to 0.1 and 0.1 to 0.5 indicated weak, intermediate, and strong similarity between accessions, respectively [35]. The GD, heterozygosity, and PIC were calculated in PowerMarker V3.25 [55], with heterozygosity being defined as the proportion of heterozygous loci detected in a single accession, and GD being defined as the probability that two alleles randomly chosen from the test sample were different [18]. The PIC was estimated as follows: ${\mathrm{PIC}}_l=1-{\displaystyle {\sum}_{u=1}^k{\tilde{\mathrm{p}}}_{\mathrm{lu}}^2}-{\displaystyle {\sum}_{u=1}^{k-1}{\displaystyle {\sum}_{v=u+1}^k{2\tilde{\mathrm{p}}}_{\mathrm{lu}}^2}}{\tilde{\mathrm{p}}}_{\mathrm{lv}}^2,$ where p_lu_ and p_lv_ are the frequencies of the *u*th and *v*th alleles for marker *l*, respectively. The LD level between SNPs was evaluated using the squared Pearson correlation coefficient (r^2^) between vectors of SNP alleles according to a previous study [56]. This evaluation was completed in the TASSEL software 5.0 [50]. The D_EST_ values were calculated following the algorithm described by Yang [57] using the R ‘hierfstat’ package (http://cran.r-project.org/web/packages/hierfstat). The D_EST_ was defined as D_EST_ = [(H_Test_-H_Sest_)/(1-H_Sest_)]/[n/(n-1)], where H_Sest_ is the observed gene diversity within subpopulation, H_Test_ is the overall gene diversity, and n is the number of subpopulations [27]. The size of genomic regions was defined as window size with double the value of the LD level of this panel, and the average D_EST_ of all SNPs in LD was defined as the cutoff value to interpret the relevant differentiation level between subpopulations. Genomic regions with an average D_EST_ exceeding the cutoff value were treated as cases of strong differentiation between subpopulations.

### Genome wide association study

The BLUP of FT-related traits, including the DTT, DTP and DTS for each accession across three environments, and 43,252 SNPs were selected to perform a phenotype-genotype GWAS, which was implemented in the GAPIT R package [58], using mix linear model (MLM) in which the population structure and pair-kinship were treated as covariates [38]. The significant cutoff value was defined as 0.05 divided by the number of markers. QTNs were selected for further analysis when the *P*-values of SNPs were less than the cutoff value.

### Availability of supporting data

All data sets supporting the results of this article are included within the article.

## Additional files

Additional file 1:
**Figure S1.** Correlation analysis of the first five principal components (PCs) between specific subset with different marker size and the entire set with all markers. **Figure S2.** Pair-comparison of D_EST_ on different chromosomes between the subpopulations. **Figure S3.** Summary statistic of flowering time related traits. Histograms show the distribution of flowering time related traits. Numbers in the up-right triangle are the Pearson coefficient between flowering time related traits and between environments for the same trait. The low-left triangle is the scatter plots between flowering time related traits and between environments for the same trait. **Figure S4.** Comparison of the genetic variation evaluated by using two datasets including 43,252 and 85 SNPs, respectively. Each scatter presents one accession. Black line is the fitted of all scatters. (ZIP 850 kb) Additional file 2:
**Table S1.** Summary information of sources and co-ancestral membership of 1857 accessions **Table S2.** GWAS results of flowering time related traits and relevant linked QTL or genes. **Table S3.** Genomic regions with D_EST_ of more than 0.2 and relevant linked QTL. (XLSX 299 kb)

## Abbreviation

SNP: Single-nucleotide polymorphisms
HZS: Huangzaosi
TS: Tropical/subtropical
FT-: Flowering time related traits
GWAS: Genome-wide association study
QTL: Quantitative trait loci
SS: Iowa Stiff Stalk Synthetic
NSS: Non-Stiff Stalk
IDT: Iodent
GBS: Genotyping by sequencing
PC: Principal components
∆K: delta K
MICN: Modified Introduction in China
LD: Linkage disequilibrium
GDs: Gene diversities
PICs: Polymorphism information contents
DTT: Days to tasseling
DTS: Days to silking
and DTP: Pollen-shedding
MLM: Mix linear model
IBS: Identity by state
BLUP: Best linear unbiased predictor
GLM: General line model
