# Supplementary figures and images for: Analysis of genetic differentiation and genomic variation to reveal potential regions of importance during maize improvement
Source: BMC Plant Biol. 2015 Oct 24;15:256. doi: 10.1186/s12870-015-0646-7 (PMC4620006; doi:10.1186/s12870-015-0646-7)

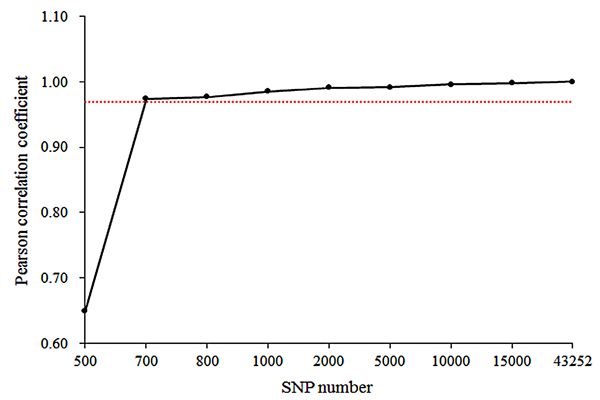

Supplement: Additional file 1: — Figure S1. Correlation analysis of the first five principal components (PCs) between specific subset with different marker size and the entire set with all markers. Figure S2. Pair-comparison of DEST on different chromosomes between the subpopulations. Figure S3. Summary statistic of flowering time related traits. Histograms show the distribution of flowering time related traits. Numbers in the up-right triangle are the Pearson coefficient between flowering time related traits and between environments for the same trait. The low-left triangle is the scatter plots between flowering time related traits and between environments for the same trait. Figure S4. Comparison of the genetic variation evaluated by using two datasets including 43,252 and 85 SNPs, respectively. Each scatter presents one accession. Black line is the fitted of all scatters. (ZIP 850 kb) [file 12870_2015_646_MOESM1_ESM.zip › Figure S1.tif]

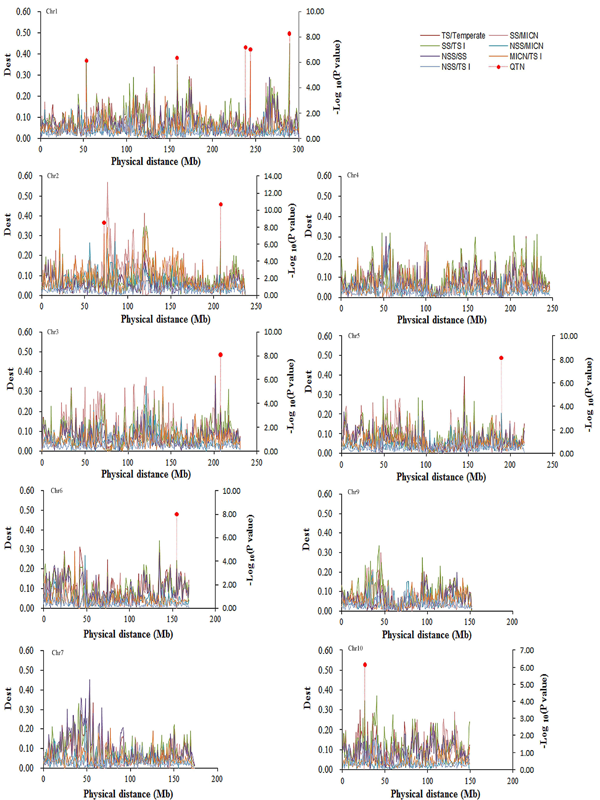

Supplement: Additional file 1: — Figure S1. Correlation analysis of the first five principal components (PCs) between specific subset with different marker size and the entire set with all markers. Figure S2. Pair-comparison of DEST on different chromosomes between the subpopulations. Figure S3. Summary statistic of flowering time related traits. Histograms show the distribution of flowering time related traits. Numbers in the up-right triangle are the Pearson coefficient between flowering time related traits and between environments for the same trait. The low-left triangle is the scatter plots between flowering time related traits and between environments for the same trait. Figure S4. Comparison of the genetic variation evaluated by using two datasets including 43,252 and 85 SNPs, respectively. Each scatter presents one accession. Black line is the fitted of all scatters. (ZIP 850 kb) [file 12870_2015_646_MOESM1_ESM.zip › Figure S2.tif]

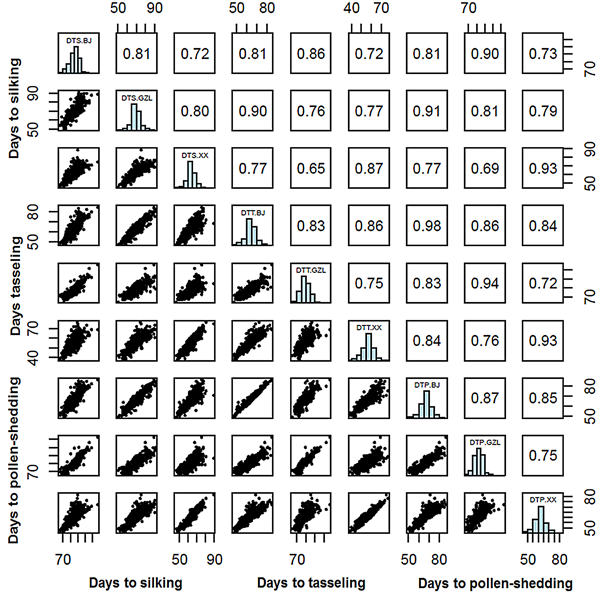

Supplement: Additional file 1: — Figure S1. Correlation analysis of the first five principal components (PCs) between specific subset with different marker size and the entire set with all markers. Figure S2. Pair-comparison of DEST on different chromosomes between the subpopulations. Figure S3. Summary statistic of flowering time related traits. Histograms show the distribution of flowering time related traits. Numbers in the up-right triangle are the Pearson coefficient between flowering time related traits and between environments for the same trait. The low-left triangle is the scatter plots between flowering time related traits and between environments for the same trait. Figure S4. Comparison of the genetic variation evaluated by using two datasets including 43,252 and 85 SNPs, respectively. Each scatter presents one accession. Black line is the fitted of all scatters. (ZIP 850 kb) [file 12870_2015_646_MOESM1_ESM.zip › Figure S3.tif]

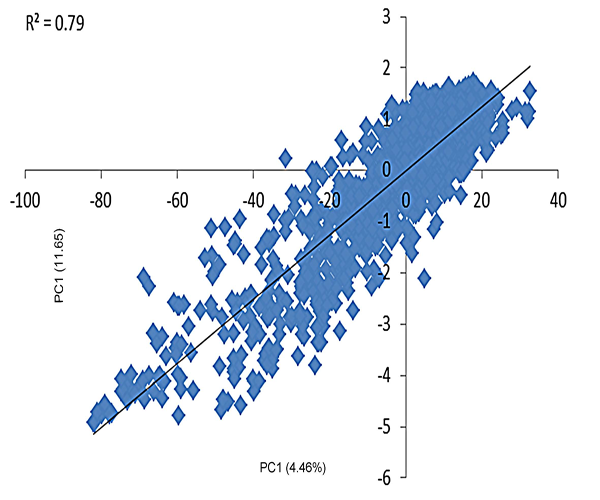

Supplement: Additional file 1: — Figure S1. Correlation analysis of the first five principal components (PCs) between specific subset with different marker size and the entire set with all markers. Figure S2. Pair-comparison of DEST on different chromosomes between the subpopulations. Figure S3. Summary statistic of flowering time related traits. Histograms show the distribution of flowering time related traits. Numbers in the up-right triangle are the Pearson coefficient between flowering time related traits and between environments for the same trait. The low-left triangle is the scatter plots between flowering time related traits and between environments for the same trait. Figure S4. Comparison of the genetic variation evaluated by using two datasets including 43,252 and 85 SNPs, respectively. Each scatter presents one accession. Black line is the fitted of all scatters. (ZIP 850 kb) [file 12870_2015_646_MOESM1_ESM.zip › Figure S4.tif]
